# Experimental validation of a kV source model and dose computation method for CBCT imaging in an anthropomorphic phantom

**DOI:** 10.1120/jacmp.v17i4.6021

**Published:** 2016-07-08

**Authors:** Yannick Poirier, Mauro Tambasco

**Affiliations:** ^1^ Department of Medical Physics Tom Baker Cancer Centre Calgary AB Canada; ^2^ Department of Physics San Diego State University San Diego CA USA; ^3^ Department of Oncology University of Calgary Calgary AB Canada

**Keywords:** cone‐beam CT (CBCT), absorbed dose, X‐ray source modeling, Monte Carlo

## Abstract

We present an experimental validation of a kilovoltage (kV) X‐ray source characterization model in an anthropomorphic phantom to estimate patient‐specific absorbed dose from kV cone‐beam computed tomography (CBCT) imaging procedures and compare these doses to nominal weighted CT‐dose index (${\text{CTDI}}_{w}$) dose estimates. We simulated the default Varian on‐board imager 1.4 (OBI) default CBCT imaging protocols (i.e., standard‐dose head, low‐dose thorax, pelvis, and pelvis spotlight) using our previously developed and easy to implement X‐ray point‐source model and source characterization approach. We used this characterized source model to compute absorbed dose in homogeneous and anthropomorphic phantoms using our previously validated in‐house kV dose computation software (kVDoseCalc). We compared these computed absorbed doses to doses derived from ionization chamber measurements acquired at several points in a homogeneous cylindrical phantom and from thermoluminescent detectors (TLDs) placed in the anthropomorphic phantom. In the homogeneous cylindrical phantom, computed values of absorbed dose relative to the center of the phantom agreed with measured values within ≤2% of local dose, except in regions of high‐dose gradient where the distance to agreement (DTA) was 2 mm. The computed absorbed dose in the anthropomorphic phantom generally agreed with TLD measurements, with an average percent dose difference ranging from 2.4%±6.0% to 5.7%±10.3%, depending on the characterized CBCT imaging protocol. The low‐dose thorax and the standard dose scans showed the best and worst agreement, respectively. Our results also broadly agree with published values, which are approximately twice as high as the nominal ${\text{CTDI}}_{w}$ would suggest. The results demonstrate that our previously developed method for modeling and characterizing a kV X‐ray source could be used to accurately assess patient‐specific absorbed dose from kV CBCT procedures within reasonable accuracy, and serve as further evidence that existing ${\text{CTDI}}_{w}$ assessments underestimate absorbed dose delivered to patients.

PACS number(s): 87.57.Q‐, 87.57.uq, 87.10.Rt

## I. INTRODUCTION

Cone‐beam computed tomography (CBCT) is commonly used in image‐guided radiation therapy (IGRT) to localize tumors or organs at risk and verify the patient position prior to treatment delivery. While the patient‐specific absorbed dose deposited by megavoltage (MV) therapeutic and imaging beams can be routinely calculated using commercially available treatment planning software (TPS), no equivalent commercial software currently exists for kilovoltage (kV) energy X‐rays which are most commonly used in imaging applications such as CBCT. The American Association of Physicists in Medicine (AAPM)'s Task Group 75 report raised awareness regarding the imaging dose accrued by patients during IGRT,^(1)^ and there has been an increasing interest in developing a fast and accurate method to compute the patient‐specific absorbed dose from kV imaging procedures and to possibly incorporate it in the radiation treatment plan for a patient.

Recent attempts to develop such a method include the adaptation of the Pinnacle TPS (Philips Medical Systems, Milpitas, CA) for kV dose computation^(2)^ and conventional Monte Carlo (MC) simulations to estimate the kV CBCT dose in a prostate patient.^(3)^ The former approach is relatively easy to implement in the clinic since it uses existing commercially available software and equipment. Unfortunately, the method of scaling energy‐deposition kernels with electron density underestimates the photoelectric effect and attenuation in bone by a factor of 3 to 4.^(4)^ In contrast, MC simulation techniques are very accurate, but are computationally intense and require specialized software implementation that make them less feasible for routine clinical use.

Kouznetsov and Tambasco^(5)^ have developed an in‐house software (kVDoseCalc) that allows one to use CT image data to rapidly and accurately compute the absorbed kV X‐ray dose at a series of points of interest (POIs). This method was validated computationally using the MCNP^(6)^ and EGSnrc^(7)^ MC simulation techniques.^(5)^ It was also validated experimentally for therapeutic kV x‐ray beams,^8^, ^9^ radiographic imaging procedures using open beams,^(10)^ diagnostic CT scanners,^(11)^ and radiographic beams produced with added bowtie filters.^(12)^ A machine‐specific virtual point source model was developed by Poirier et al.^10^, ^12^ to simulate the X‐ray beam input. This source model is characterized from simple in‐air ionization chamber measurements without the need for specialized equipment or knowledge of MC modeling techniques.

However, in previous studies the dose computation method was only validated for stationary radiographic imaging beams using reference field sizes of $20\times 20\;{\text{cm}}^{2}$. In contrast, CBCT beams are sometimes asymmetrical to provide increased field of view. The purpose of this study is to provide experimental validation of the method for a fully characterized rotating CBCT source (Varian On‐Board Imager (OBI) 1.4 imaging unit, Varian Medical Systems, Inc, Palo Alto, CA) using a cylindrical and an anthropomorphic phantom.

To provide increased confidence in our results, the doses measured in the anthropomorphic phantom are compared to doses estimated from nominal weighted CT Dose Index (${\text{CTDI}}_{w}$) values.^(13)^ Additionally, the measured doses are compared to those obtained by Palm et al.^(14)^ in a similar anthropomorphic phantom imaged using the same CBCT imaging protocols.

## II. MATERIALS AND METHODS

### A. Model and calculation overview

#### A.1 Software algorithm overview

All computed absorbed dose values were calculated by kVDoseCalc, a kV dose computation software developed in‐house by Kouznetsov and Tambasco.^(5)^ The software computes the dose by numerically evaluating the linear Boltzmann transport equation (LBTE) to find the differential photon angular flux density at a point of interest (POI), and converts this to absorbed radiation dose using collisional‐kerma factors based on the coherent and incoherent angular distribution functions and photoelectric absorption interaction cross sections.

The differential angular flux density is separated into its primary and scattered components. The primary component of the differential angular flux density is calculated deterministically using exponential attenuation and the beam divergence as described by the inverse‐square law. The scattered component is further separated into a first‐ and a multiple‐collision contribution, which are calculated using biased MC methods to generate a population of representative scattering points. The scatter component of the differential angular flux density at the POI is calculated by using these scattering points as a secondary radiation source in the numerical evaluation of the LBTE.^(5)^ To evaluate the interaction cross sections throughout the medium, the software requires a DICOM‐format CT image in which Hounsfield units (HU) ranges are assigned a physical density ($g/{\text{cm}}^{3}$) and an elemental material composition ($\text{atoms}/{\text{cm}}^{3}$). The interaction cross sections from the ENDF/B‐VI microcross‐sectional library^(15)^ are used to calculate total interaction cross sections of each material. These interaction cross sections are used to compute the optical length of the photon trajectories required for the photon transport for the various components of the differential angular flux density. The dose is computed from the flux (the differential angular flux density integrated over all solid angles) through flux‐tokerma conversion factors which are also precalculated for each photon energy and material composition in the CT image by integrating the differential distribution of photon energy fluence with the mass energy‐transfer coefficient of each possible energy and scattering angle.^(5)^

#### A.2 Source model

The kV X‐ray source is modeled by a virtual point source created from the user‐specified spatially varying spectrum and planar fluence at the isocenter plane. This virtual point source is used to create the photons which are transported in the biased MC calculations of the scattered component of the differential angular flux density. In this model, photons are generated with a given energy and trajectory from the source location (the focal spot on the X‐ray tube anode) to the isocenter plane.^(12)^ To model rotating CBCT sources, the software was modified to incorporate a starting gantry angle and arc size. Photons are assigned a random angle in this angular range to assign random origins to each photon.

The dose computations were performed at each POI using 1.5 million seeded photons on four 3.20 GHz Intel Core 7 960 CPU (Intel Corp., Santa Clara, CA). The virtual point source was placed at a 100 cm surface‐to‐axis distance (SAD) from the isocenter for all computations so as to match the default CBCT imaging parameters of the Varian OBI 1.4 imaging system.

#### A.3 CBCT source characterization

**Figure 1:**
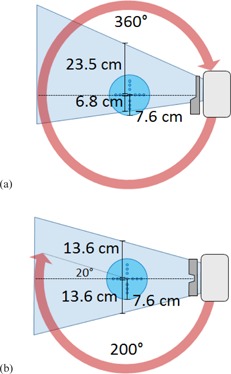
Imaging geometry for Varian OBI 1.4 CBCT default technique settings. The pelvis and low‐dose thorax (a) techniques use an asymmetric imaging field and a full 360° arc, while the high‐quality head and pelvis spotlight (b) techniques use a symmetric imaging field and a partial 200° arc. The homogeneous cylindrical acrylic phantom (r=7.6 cm) from Fig. 2 is shown for reference.

The X‐ray source of a Varian OBI 1.4 imaging unit was characterized for four default kV CBCT imaging protocols (Table 1, Fig. 1) using a previously validated kV source characterization method and virtual point source model.^(12)^ Briefly, this method involves measuring the half‐value layer (HVL) along the transverse axis and the in‐air kerma profiles along the radial and transverse axes. The spatially varying spectrum and fluence of the source are then derived from these values.

**Table 1:** Default settings for CBCT procedures using the Varian OBI 1.4 system

*CBCT Protocol*	*Standard‐dose Head*	*Pelvis Spotlight* ^a^	*Low‐dose Thorax*	*Pelvis*
*Filter*	*Full‐bowtie*		*Half‐bowtie*	
Field size (cm)	$\left(x_{1},x_{2})=(13.6,13.6)\;(y_{1},y_{2})=(9.2,9.2\right)$		$\left(x_{1},x_{2})=(6.8,23.5)\;(y_{1},y_{2})=(10.3,10.3\right)$	
Arc size	200°		360°	
Peak energy (kVp)	100	125	110	125
Exposure (mAs)	145	720	262	580

a ^a^ The pelvis spotlight could be used either with the full‐ or half‐bowtie filter.

**Figure 2:**
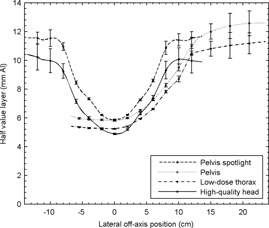
Characterization of CBCT imaging protocol spectra through measurement of HVL. Points represent measured values and lines indicate interpolated values. The pelvis and low‐dose thorax protocols use an asymmetric field and a half‐bowtie filter, while the pelvis spotlight and high‐quality head protocols use a symmetric field and a full‐bowtie filter, which explains their characteristic shapes.

**Figure 3:**
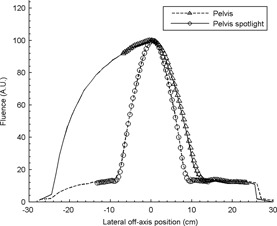
Fluences of CBCT imaging beams. Points represent values interpolated from measurements while lines represent values obtained from Monte Carlo by Ding et al.^(4)^ The values obtained empirically are in excellent agreement with theoretical values, except at the point of infection where the bowtie filters become flat where the spline interpolation causes ripples. Low‐dose thorax and high‐quality head fluencies are not depicted since they are nearly identical to their higher‐energy counterparts, and no theoretical values are available.

The in‐air kerma and HVL were measured at 2 cm intervals for all four beam qualities using a 0.65 cc PR‐06C Capintec Farmer‐style chamber (Capintec Inc., Ramsey, NJ) calibrated in the kV energy range (60, 80, 100, 150, and 200 kVp) and a Standard Imaging SuperMAX electrometer (Standard Imaging, Middleton, WI) set in the low‐dose sensitivity range with an applied potential bias of +300 V. Figures 2 and 3 report the characterized values of HVL (directly related to spectrum) and fluence, respectively.

We chose to only characterize the pelvis, pelvis spotlight, low‐dose thorax, and high‐quality head default Varian OBI 1.4 CBCT protocols, leaving out the normal‐quality and low‐dose head protocols. Since these additional protocols were identical to the high‐quality head (except in mAs), only one head CBCT protocol was characterized. We obtained the parameters for each technique from official Varian documentation. The ${\text{CTDI}}_{w}$, or weighted CT dose index, is a weighted average of five dose measurements in a homogeneous cylindrical phantom using a long (10 cm) ionization chamber. Nominal values for the ${\text{CTDI}}_{w}$ are provided by the manufacturer (reported in Table 1).

Of note, we chose to characterize the pelvis spotlight protocol using a full‐bowtie filter as opposed to the half‐bowtie filter, as Varian suggests that doing so reduces the ${\text{CTDI}}_{w}$. Furthermore, it allowed us to directly compare our results with similar measurements reported by Palm et al.^(14)^ where the full‐bowtie filter was used.

### B. Validation

We irradiated two phantoms using the characterized OBI 1.4 CBCT default imaging protocols to validate our approach. To provide validation in homogeneous and heterogeneous patient‐mimicking geometries, we used a cylindrical acrylic phantom similar to a CTDI head phantom and an anthropomorphic phantom, respectively.

#### B.1 Cylindrical homogeneous phantom measurements

**Figure 4:**
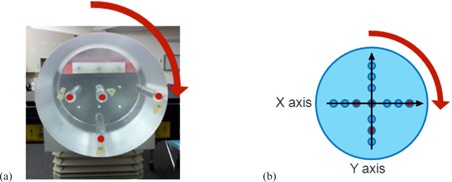
Cylindrical acrylic phantom (a) with dots showing possible ionization chamber positions at radii r=0, 3.6, 5.2, and 6.6 cm, respectively. The phantom can be rotated in increments of 90° which allows dose measurements along the x‐ and y‐axes (b).

Holes were drilled to accommodate the Capintec 0.65 cc ionization chamber at the center and at three different radial positions in a 15.2 cm diameter acrylic phantom that was constructed in house (Fig. 4(a)). The phantom is designed to rotate in 90° increments to enable dose measurements at 13 different positions (Fig. 4(b)). A fitted acrylic sleeve accommodates the ionization chamber at the measurement points of interest, while the other holes are filled with whole acrylic rods.

The center of the acrylic phantom was placed on a support so as to hang at the end of the couch to insure that the beam would not be attenuated by the couch. We positioned the phantom at the isocenter of the beam using the in‐room patient positioning lasers (calibrated to within 1 mm accuracy) and acquired an image at each characterized CBCT setting (Table 1). The dose was measured at each of the 13 points consecutively for all CBCT settings. To assess the error due to positioning and output fluctuations, we repeated the measurements over three days and found that the measurements were reproducible within ±0.8%.

#### B.2 Cylindrical homogeneous phantom calculations

We constructed idealized DICOM images matching the physical dimensions of our homogeneous cylindrical phantom. The image was composed of 512×512×91 voxels (~23.8 million voxels) with dimensions of $0.81\times 0.81\times 3\;{\text{mm}}^{3}$ in the X, Y, and Z directions, respectively. The phantom it represented was fabricated with acrylic material by our machine shop with a diameter of 15.2 cm and a length of 22 cm (see Materials & Methods section B.1 above). We used the nominal acrylic molecular composition C_5_O_2_H_8_ and the measured physical density of $1.16g/{\text{cm}}^{3}$ to calculate the atomic number density ($\text{atom}/{\text{cm}}^{3}$) for each element required by kVDoseCalc. We computed profiles along the x‐ and y‐axes (Fig. 4(b)).

#### B.3 Anthropomorphic phantom measurements

**Figure 5:**
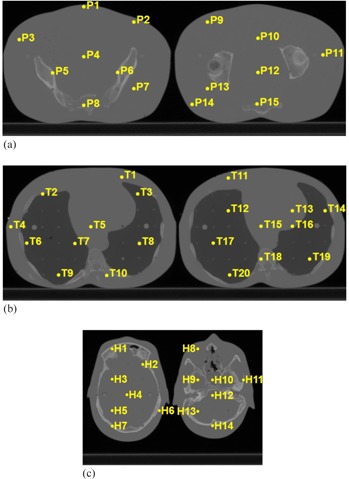
Measurement and computation points inside the anthropomorphic RANDO phantom in the pelvis (a), the thorax (b), and the head (c). Points are numbered from left to right, top to bottom.

We measured the absorbed dose inside the anthropomorphic RANDO phantom (The Phantom Laboratory, Salem, NY) using high‐sensitivity MCP‐N (LiF:Mg,Cu,P) TLD chips (Radcard, Krakow, Poland). We positioned the 4.5 mm diameter and 1 mm width TLD chips in the predrilled holes in the RANDO phantom. They were held in place by two small acrylic cylinders positioned in a way that sandwiched the chips between them, minimizing any potential air gaps. The locations of the TLDs (Fig. 5) were chosen to sample as many positions and tissues as possible within the two central slices of the relevant anatomical site (i.e., pelvis, thorax, or head). The sites were imaged using the appropriate default CBCT imaging technique (Table 1). We positioned the phantom by aligning the in‐room positional lasers to external markers recognizable on the CT image used in the dose computation.

#### B.4 Anthropomorphic phantom calculations

We computed absorbed dose inside the anthropomorphic RANDO phantom comprising tissue‐ and lung‐equivalent materials, and human bones. We imaged the RANDO phantom using a Brilliance Big Bore CT scanner (Phillips Healthcare, Best, The Netherlands) and imaging protocols standard at our institution: the head (512×512×191 voxels of size $0.70\times 0.70\times 2.5\;{\text{mm}}^{3}$) and body (512×512×236 voxels of size $0.81\times 0.81\times 3\;{\text{mm}}^{3}$) scans. The CT image was exported in DICOM format to use in kVDoseCalc. We assigned material compositions and physical densities to ranges of HU values (cf., Table 2).

Bone composition varies according to the anatomical site (e.g., femurs in pelvis), so we only implemented the relevant bone types for the relevant sites. The manufacturer provided the nominal material compositions (expressed in mass percentage) and physical densities for the RANDO tissue‐ and lung‐equivalent materials. We used published values from the International Commission on Radiation Units and Measurements (ICRU) Report 46^(16)^ for the various bone material compositions and physical densities assuming adult bones. While the number of photons seeded is sufficient to lower the statistical uncertainty of our computation to below 1%, the TLDs have a finite volume within which the dose may vary. We computed the absorbed dose over a uniform distribution of POIs corresponding to the physical size of the detectors (3 pixel radiusã2.46 mm and 1pixel length=3 mm). The uncertainty due to the dose gradient over the detector measurement volume was assessed by the standard deviation of the average dose of these points.

**Table 2:** HU to materials conversion table

*Imaged Location*	*HU Range*	*Material*	*Physical Density* ($g/{\text{cm}}^{3}$)
Pelvis	‐1000–‐300	Air	0.00120
	‐300–‐50	RANDO (tissue)	0.997
	5–100	Bone marrow	1.03
	100–400	Femur	1.33
	400–10 000	Cortical bone	1.92
Thorax	‐1000–‐750	Air	0.00120
	‐750–‐30	RANDO (lung)	0.352
	‐300–‐50	RANDO (tissue)	0.997
	50–10 000	Cortical bone	1.92
Head	‐1000–‐300	Air	0.00120
	‐300–50	RANDO (tissue)	0.997
	50–10 000	Cortical bone	1.92

#### **B.5 *Absorbed dose calibration***

The output of the Varian OBI 1.4 imaging unit was evaluated using the in‐phantom method outlined by the AAPM's Task Group 61 (TG‐61)^(17)^ guidelines on kilovoltage energy dosimetry. We used the same ionization chamber and electrometer used to characterize the X‐ray beams. The ionization chamber was placed at a depth of 2 cm in Gammex Solid Water (Gammex Inc.. Middleton, WI) with 6 cm of backscatter. The outputs corresponding to a calibration exposure of 160 mA and 160 ms (25.6 mAs) were measured for a calibration field ($20\times 20\;{\text{cm}}^{2}$) and the relevant CBCT fields (Table 1) for each energy and filter combination. We verified that the output linearity of the imaging unit was within 1% for the relevant imaging exposures by measuring the dose with varying currents and time settings.

#### **B.6 *TLD characterization***

According to the manufacturer, MCP‐N TLD chips exhibit a sensitivity 30 times greater than conventional MTS‐N (LiF:Mg,Ti) TLDs and have a detection threshold of 50 nGy making them ideal for measuring the low absorbed doses deposited by CBCT imaging procedures. Furthermore, while conventional MTS‐N TLDs exhibit an energy response limiting their use in kV dosimetry applications, Duggan et al.^(18)^ showed that MCP‐N TLDs are not significantly energy‐dependent at low energies.

TLD chips can only be read once before they must be annealed to restore their sensitivity. Following the manufacturer's guidelines, the chips were annealed at 100°C following an irradiation to eliminate noise due to low‐energy electron traps. The TLDs were read by a photomultiplier tube (PMT) at a temperature of 240°C, after which they were annealed at a temperature of 240°C. Each annealing was followed by a rapid cooling by placing the chips directly on an aluminum surface.

To assess the reproducibility of the TLD chips, they were irradiated five times at the center of a $20\times 20\;{\text{cm}}^{2}$ beam in a phantom of therapy‐grade Solid Water (Gammex Inc.) at a depth of 2 cm and with 6 cm of backscatter. The phantom was placed at a source‐to‐surface distance (SSD) of 100 cm and irradiated with a technique setting of 160 mA and 160 ms (25.6 mAs) using the relevant energies and corresponding bowtie filter. The response of the TLD chips stabilized after 3–5 irradiation/annealing cycles. We observed that the average sensitivity of the TLD batch could vary from one exposure to the next, but that the response of an individual chip against the average of the whole never varied by more than 3%. Therefore, three chips were kept aside during each experiment and exposed using the calibration dose to eliminate the uncertainty related to variations in sensitivity between annealing cycles. Once stabilized, the standard deviation of five consecutive exposures was used to assess the reproducibility of the TLD readings, and was found to range from 1%–3%. The dose response of each TLD chip was individually determined over a range of three different exposures (mAs) by a linear fit of the measured charge (Q) and the absorbed dose (D), as shown by the following expression,

(1) D=mQ+b

where *m* and *b* represent the slope and intercept of the linear fit, respectively. Values for the absorbed dose D were obtained by ionization measurements under the same conditions. The uncertainty in the absorbed dose ΔDi was estimated by the expression

(2) ΔD=QΔm+mΔQ+Δb

where *Δm* and *Δb* represent the TLD‐specific fit uncertainties of the slope and intercept, respectively, and *ΔQ* represents the uncertainty in measured charge, where ΔQ/Q is the normalized standard deviation of the TLD readings discussed earlier. These characterizations were carried out for each energy shown in Table 1.

#### B.7 Tissue conversion factors

At low kV energies, electrons have a maximum range on the order of micrometers, so they can be considered to be locally absorbed.^5^, ^19^ Therefore, the TLDs act as photon detectors; the measured TLD dose depends only on the incident photon fluence, and no tissue‐specific calibration is required when the chips are placed in the RANDO tissue and lung‐equivalent materials. Since the chips were characterized in Gammex Solid Water, they report absorbed dose‐to‐tissue‐equivalent material, signifying that tissue‐equivalent dose was measured and not the dose‐to‐bone or lung‐equivalent materials.

However, unlike many dose computation algorithms, kVDoseCalc calculates dose to the specific material instead of the dose to water. In the thorax, the spacing plugs are comprised of RANDO lung‐equivalent material. To compare equivalent qualities, we determined factors to convert calculated dose‐to‐lung to calculated dose‐to‐tissue. There was no need to account for bone‐to‐tissue conversion factors, as standard RANDO tissue‐equivalent spacing plugs are present in every region outside the lungs, including bony regions. We obtained this lung‐to‐tissue conversion factor empirically by using kVDoseCalc to compute the dose in a voxel located in a cylinder of lung‐equivalent material, then replacing that same voxel with a tissue‐equivalent voxel, and using the ratio of both quantities as the conversion factor.

As kVDoseCalc simulates particle histories, this conversion factor is based on the spectrum at the position of the TLD, which has been hardened and attenuated by interactions in the phantom. However, due to the rotation of the CBCT beam causing photons to come in from varying directions, and the low‐Z nature of both tissue and lung, the variations of the conversion factor are within ±1.2% for all the points we investigated.

## III. RESULTS

### A. Cylindrical acrylic phantom

**Figure 6:**
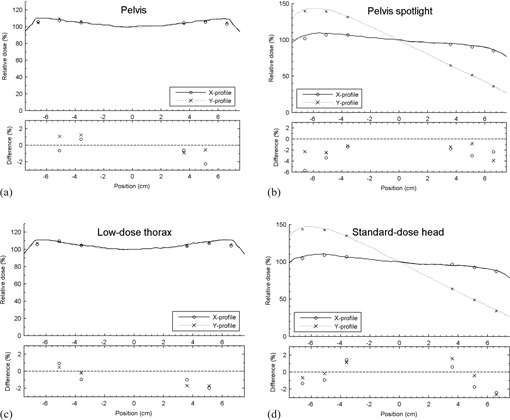
Comparison (a) to (d) between measured (circles and crosses) and computed dose (lines) relative to the center of the cylindrical acrylic phantom for each imaging protocol shown in Table 1. Axes are defined according to Fig. 1. Positive differences mean that measurements are higher than calculations and vice‐versa.

The agreement between ionization chamber measurements and the dose computed with kVDoseCalc for the cylindrical acrylic phantom was within 2% of the local dose for most points (Fig. 6). There was an abrupt dose dropoff at a radius of 6.8 cm in the pelvis and low‐dose thorax beams (Figs. 6(a) and (c)) due to the asymmetric field size of 6.8−23.5 cm in the transverse axis for these CBCT protocols.

For the head and pelvis spotlight beams, scans (Figs. 6(b) and (d)), the measurements agreed with computation to within 2% of local dose for the standard‐dose head scan and within 4% for the pelvis spotlight scan. As these CBCT techniques use a 200° arc, the anterior portion of the phantom (+Y direction) only receives exit dose, whereas the posterior portion receives only entrance dose (see Fig. 1(b)). There is therefore a dependence in the Y direction absent from the CBCT techniques using a full 360° arc.

### B. Anthropomorphic phantom

**Figure 7:**
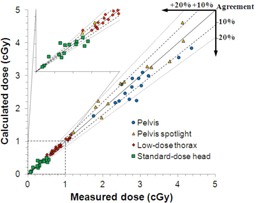
Comparison between measured and computed dose inside the anthropomorphic phantom for all imaging technique settings. The full line indicates a perfect agreement (slope=1), while the dotted lines indicate discrepancies ranging from ‐20%−20%.

**Figure 8:**
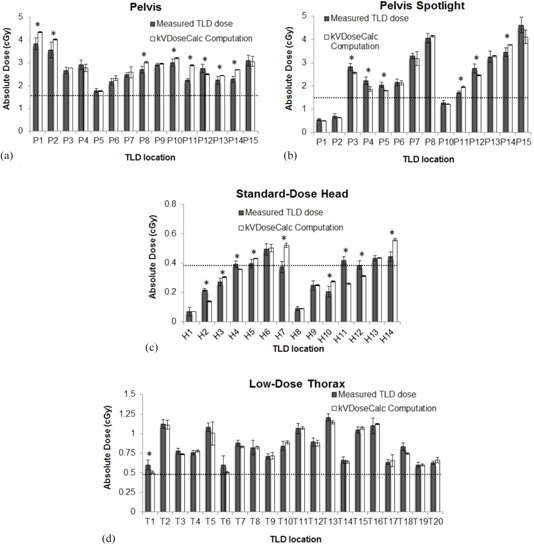
Comparison between measured absorbed TLD dose (white columns) and kVDoseCalc computations (grey columns) for the pelvis (a), pelvis spotlight (b), low‐dose thorax (c), and standard‐dose head (d) CBCT default parameters. The error bars of the measured values generally overlap with those of the computed values. Points for which the error bars do not overlap are marked with an asterisk (*). The dotted line indicates the nominal CTDIw dose for that imaging protocol as a comparison. The results are further summarized in Table 3.

We compared TLD dose measurements to kVDoseCalc computation in Figs. 7 and 8. Figure 7 shows that the calculated dose correlates very well with measured values. The local percent difference is usually within 10%, and always within 20%, except in the head and a single point for the pelvis protocol.

We calculated the average and standard deviation of the percent difference in local absorbed dose between measurement and computation, and found that it varied between 2.4%±6.0% and 5.7%±10.3% according to the site and imaging protocol. The low‐dose thorax exhibited the best agreement with an average of ‐2.4% and a standard deviation of 6.0%, and with the measured and computed dose error bars overlapping for every point (i.e., agreement within experimental uncertainty). The thorax was also the imaging site exhibiting the least variation in structures as the lung‐equivalent material is fairly homogeneous, and the ribs and spine are the only significant bony structures. Conversely, the standard‐dose head scan showed the most variation (‐4.1%±26.9%) in agreement, because this geometry exhibits the most heterogeneity (e.g., dense and shallow bones, air cavities). In contrast, the two pelvic scans (pelvis and pelvis spotlight) showed similar results (5.6%±8.3% and ‐5.7%±10.3%, respectively), which suggests that the imaging site, and consequently the HU‐to‐materials conversion, is the most important factor determining the agreement between computation and measurement.

**Figure 9:**
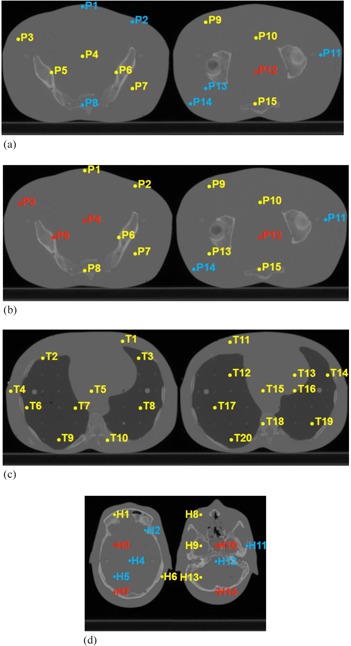
Points inside the anthropomorphic phantom where the computed and measured dose intervals of confidence do not overlap for the pelvis (a), the pelvis spotlight (b), the low‐dose thorax (c), and the head (d) scans. Yellow points show where the intervals of confidence overlap, while blue and red points indicate where the computed dose is lower or higher than TLD measurements and the error bars do not overlap, respectively.

A point‐by‐point comparison is shown in Fig. 8. While the agreement is generally good, it is not always within experimental uncertainty. We see that most of the error bars associated with the measured value overlap with those of the computed values, ranging from 36% to 100% of the values depending on the imaging protocol (Table 3). Figure 9 shows the location of the points in which error bars do not overlap.

**Table 3:** Summary of the measured, computed, and published values for dose in the anthropomorphic phantom

*CBCT Protocol*	*Pelvis*	*Low‐dose Thorax*	*Pelvis Spotlight*	*SD Head*
Measured TLD (cGy)	1.78−3.84	0.60−1.12	0.56−4.62	0.07−0.50
kVDoseCalc (cGy)	1.75−4.35	0.51−1.12	0.50−4.14	0.07−0.56
Avg. Agreement	5.6%±8.3%	‐2.4%±6.0%	‐5.7%±10.3%	‐4.1%±26.9%
Pass/fail	8/15 (53%)	20/20 (100%)	8/14 (57%)	5/14 (36%)
Nominal ${\text{CTDI}}_{w}$ ^a^ (cGy)	1.77	0.47	1.44	0.39
Palm et al.^b^ (cGy)	2.4−3.1	0.9−1.7	0.5−6.0	0.1−0.6

a ^a^ Nominal values from the Varian OBI Reference Guide.^(13)^

b ^b^ Measured TLD dose in anthropomorphic Alderson phantom by Palm et al.^(14)^

## IV. DISCUSSION

### A. Cylindrical homogeneous phantom

The percent difference between kVDoseCalc computation and measured dose relative to the center in the homogeneous cylindrical phantom is ≤2%, except in regions of high gradient for both protocols using a full 360° imaging arc (i.e., pelvis and low‐dose thorax). For these protocols, points measured at the outermost position (r=6.6 cm, see Fig. 4) are sampling the high‐gradient region of the field where the $x_{1}=6.8\;\text{cm}$ blade defines the edge of the high‐intensity side of the beam. Thus, there is a dose volume averaging effect in the 6 mm diameter ionization chamber which leads to the measured dose being lower than computation, with a distance‐to‐agreement of ≤2 mm.

The high‐quality head and pelvis spotlights (Figs 6(b) and (d)) exhibit a strong dependency between dose and the position along the y‐axis, and a smaller dependency along the x‐axis. This is caused by the partial 200° arc common to both techniques. This partial arc deposits entrance dose only in the posterior aspect of the phantom (‐y), while the anterior aspect of the phantom (+y) only receives exit dose (see Fig. 1(b)). In fact, the dose at the anterior surface of the phantom is only ~30% of the maximal dose. This large nonsymmetrical dose distribution is intentional, and meant to reduce dose to orbital structures for the head scans, and possibly to reduce overall dose for the pelvis spotlight scan,

As mentioned previously, kVDoseCalc computes the dose deposited by the primary, first scattered, and multiple‐scattered photons separately. kVDoseCalc had previously been validated only for radiographic exposures incident on flat surfaces where there were no contour effects. In contrast, this study involves a rotating beam incident on a round phantom. At the center of the phantom, about 50% of the dose is deposited by the scattered component. An accurate computation of the dose in this region, therefore, requires not only an accurate characterization of the beam and geometry, but also an accurate computation of these multiple scattering events.

Our results show that kVDoseCalc can calculate the dose delivered by a rotating beam incident on a curved homogeneous surface, with the same level of accuracy as previously achieved in flat phantom surfaces.^(12)^

### B. Anthropomorphic phantom

We compared TLD dose measurements to kVDoseCalc computations in the anthropomorphic phantom for four different default CBCT imaging protocols (Figs. 7 and 8). These comprise three different imaging locations, three energies, two bowtie filters (Table 1), and a number of different materials (e.g., RANDO tissue and lung‐equivalent materials, cranial, spine, and femoral bones). Despite the great variety in measurement conditions and a range of two orders of magnitude in the measured dose (0.07 to 4.62 cGy), we obtained excellent correlation between measured and calculated values (Fig. 7). For all four imaging protocols, the average of the local dose percent difference is close to zero, which shows that there is no obvious systematic difference between computation and measurement. While the error bars of the TLD measurements do not overlap with computed values in all cases, it is important to note that that these error bars only represent uncertainties which could be evaluated quantitatively such as TLD reproducibility and uncertainties in the linear fit in the TLD characterization. They do not include uncertainties due to CT image artifacts and tissue segmentation, which are much more difficult to estimate.

### C. Comparison with third parties

Palm et al.^(14)^ measured the CBCT dose in a similar anthropomorphic phantom imaged using the same OBI 1.4 default CBCT protocols, but in different locations. As shown in Table 3, our measurements generally fall within the same range as those of the Palm study for all CBCT scans. These results provide some further validation for our methodology.

We also confirm the findings by Palm and colleagues that absorbed doses in the anthropomorphic phantom are generally about twice as high as the nominal ${\text{CTDI}}_{w}$, except for head scans.^(14)^ This is explained by differences in sizes between a body CTDI phantom and RANDO. Indeed, the latter is ~57% smaller in volume than the former, so the beam experiences less attenuation. Conversely, the head CTDI phantom is only ~90% of the volume of RANDO's head, so the nominal ${\text{CTDI}}_{w}$ is a much better estimate of the absorbed dose. This confirms previous reports that the ${\text{CTDI}}_{w}$ is not adequate to estimate the patient‐specific absorbed dose.^20^, ^21^, ^22^ Additionally, despite the fact that the nominal value of the ${\text{CTDI}}_{w}$ in the head falls within the same order of magnitude as measured doses in the anthropomorphic phantom, it fails to account for the large (0.07−0.49 cGy) dose variations throughout the volume.

There are other methods currently being developed to estimate the patient‐specific CBCT dose, such as conventional MC algorithms^(23)^ or an adapted MV commercial TPS.^(2)^ A study by Alaei et al.^(2)^ compares TLD measurements, adapted MV TPS calculations, and conventional MC computations inside an anthropomorphic phantom. The agreement we obtained is slightly better than that obtained by adapted TPS (‐8% to 8% average percent difference), and comparable to that obtained by conventional MC modeling (0.3% to 7.9% average percent difference). Another MC validation in an anthropomorphic phantom was performed by Li et al.^(24)^ who modeled a conventional CT scanner. They found that the local difference between MC simulation values and TLD measurements ranged from ‐17.2% to 13.0%, which are comparable to our results.

The main advantage of the X‐ray beam characterization method we used is that it relies on a handful of values that can be measured using equipment available in most radiation therapy clinics. It is therefore easier to implement than modeling the beam using conventional MC simulation. While adapted MV TPS are interesting due to their clinical feasibility, they still require the measurement of many percent depth‐dose and profile curves^(2)^ which represent more measurements than required using our beam characterization method.^(12)^

The physics aspects of our in‐house dose calculation system (kVDoseCalc) had been validated computationally against MC simulation techniques.^(5)^ However, the absorbed dose in a patient can only be accurately computed if the input parameters are accurate. We have previously validated both our X‐ray source/beam characterization method and kVDoseCalc experimentally for a stationary beam incident on homogeneous and heterogeneous phantoms.^10^, ^12^ The homogeneous cylindrical phantom measurements in this work show that kVDoseCalc can accurately compute the dose deposited by a rotating X‐ray source defining a rotating beam incident on curved surfaces. Therefore, in principle one should be able to accurately compute the dose in an anthropomorphic phantom. However, computing the dose in anthropomorphic phantoms or patients introduces procedural sources of error that are not specific to kVDoseCalc, in particular the issue of accurate tissue segmentation.

### D. Tissue segmentation

Real patients and anthropomorphic phantoms contain bones in which the physical density and effective Z varies significantly due to differences in high‐Z phosphorus and calcium content.^(25)^ While soft tissues also vary in density and material composition, they do so to a far lesser extent.^(26)^

The difficulties involved in segmenting the irradiated volume into ranges of physical densities and material compositions introduce errors in both photon transport and fluence‐to‐dose conversions.^(27)^ According to a study by Zhou et al.,^(25)^ using the common three‐tissue HU‐to‐material segmentation scheme to compute the dose deposited by a 120 kVp beam can lead to errors of up to 100% in bone and up to 30% in adjacent soft tissues. They proposed a 42‐bones segmentation scheme for accuracy within 2.5% throughout the volume. In addition to the conventional HU, images taken with a dual‐energy CT scanner yield volumetric effective Z. Bazalova et al.^(28)^ have proposed a 39‐tissues segmentation scheme based on both of these quantities for even greater accuracy compared to a 4‐ or 8‐tissue segmentation scheme.

We found that the fraction of points where measured and calculated dose error bars overlapped was best in the thorax (20/20) and worst in the head (5/14). The thorax contains few complex bony structures, while the head contains a larger variation in bone HU. Thus, we suspect that the agreement between measured and calculated dose, as well as the accuracy in calculating dose in patients, could be improved by implementing a more complex tissue segmentation scheme.

### E. Imaging dose optimization

We have shown that our kV source model and dose computation method can be used to accurately calculate the absorbed dose from a CBCT scan to an anthropomorphic phantom that mimics patient geometry and tissue heterogeneity. However, this information is only useful insomuch as it can be used to make informed decisions regarding imaging dose.

In this study, we have characterized only the default Varian OBI 1.4 CBCT imaging protocols; however, there are a near‐infinite number of possible technique settings that would yield different image quality and dose distributions. Although these default imaging protocols are commonly used the clinically, they are not optimized for varying patient sizes. For instance, the pelvis spotlight CBCT mode has identical technique settings to the high‐quality head, including the partial 200° arc, except for kVp and mAs. In the case of head scans, this partial arc is meant to reduce doses to optical structures, but there are no particularly sensitive organs at risk to spare in the case of the pelvis scans. It might be possible to adjust the technique settings (e.g., full arc) for the pelvis spotlight to allow better image quality without drastically increasing patient dose.

A comprehensive study of image quality as a function of imaging dose, which is dependent on patient size, composition, and geometry, would allow for the optimization of technique settings that give the best image quality for minimal dose. In future work, we intend to use the methods presented in this study to explore this optimization.

## V. CONCLUSIONS

This study represents proof of principle of the development of a fast and accurate dose computation system with possible applications for estimating patient‐specific dose from kV CBCT imaging procedures. This absorbed dose calculation is more accurate than estimating absorbed dose to patients based on ${\text{CTDI}}_{w}$ values, which are not patient‐specific and do not represent the real imaging geometry. Further studies will focus on improving accuracy by implementing a more comprehensive tissue segmentation, and developing methods to rapidly and accurately compute absorbed dose not just to POIs, but over entire volumes.

## ACKNOWLEDGMENTS

We would like to thank Alexei Kouznetsov for his help in implementing the rotating X‐ray source model in kVDoseCalc. We would also like to thank Eduardo Villareal‐Barajas for his instrumental role in developing the characterization protocol for the TLDs.

## COPYRIGHT

This work is licensed under a Creative Commons Attribution 3.0 Unported License.
